# Novel Extended-spectrum β-Lactamase in *Shigella sonnei*

**DOI:** 10.3201/eid1304.061160

**Published:** 2007-04

**Authors:** Agnes Lefort, Guillame Arlet, Olivier F. Join-Lambert, Marc Lecuit, Olivier Lortholary

**Affiliations:** * Hôpital Necker-Enfants Malades, Université Paris V, Paris, France; † Centre d'Infectiologie Necker-Pasteur, Paris, France; ‡ Unité de Formation et de Recherche de Médecine Pierre et Marie Curie, Université Paris VI, Paris, France

**To the Editor:** A 38-year-old French man with a history of chronic juvenile arthritis was referred to the Necker-Enfants Malades University hospital (Paris, France) with a dysenteric syndrome. The patient had returned the day before from a 1-month stay in Port-au-Prince, Haiti, where he spent most of his time in close contact with young children from an orphanage, most of whom had diarrhea. Clinical examination at admission showed fever (39°C), chills, diffuse abdominal pain, bloody diarrhea, and vomiting. The patient received ceftriaxone, which was stopped on day 4 because initial blood and stool cultures were negative for pathogens and clinical signs had completely resolved.

Ten days later, he reported the recurrence of diarrhea without fever. A novel stool culture grew *Shigella sonnei*. An extended-spectrum β-lactamase (ESBL) was detected by double-disk synergy test; the isolate was also resistant to aminoglycosides (except amikacin), tetracycline, and cotrimoxazole. The strain was susceptible to fluoroquinolones and fosfomycin. It also appeared susceptible to azithromycin (MIC 4 μg/mL), although azithromycin MIC for *Shigella* spp. should be interpreted with caution (*1*). The patient was successfully treated with azithromycin at a dose of 500 mg/d for 5 days. Azithromycin was preferred to fluoroquinolones to avoid the risk for tendinopathy because of the patient’s history of chronic juvenile arthritis and because this antimicrobial agent was shown to be effective in the treatment of shigellosis caused by multidrug-resistant strains (*2*).

To identify the molecular basis of this ESBL, a series of PCR primers were used for detection of TEM-, SHV-, or CTX-M–type ESBL (*3*). Only the TEM PCR showed positive results. Sequencing of 2 independent PCR products showed a new allele (www.lahey.org/studies/temtable.asp). Analysis of the deduced amino acid sequence allowed characterization of TEM-137, derived from TEM-1 with 2 substitutions, Arg-16→Ser and Glu-240→Arg. This ESBL (and resistance to aminoglycosides and tetracyclines) was easily transferred to *Escherichia coli* J53-2 by conjugation.

MICs of β-lactams alone or in association with clavulanic acid, were determined by E-test, according to manufacturer’s instructions (AB Biodisk, Solna, Sweden). High-level resistance to ceftazidime (MIC 32 μg/mL) and intermediate resistance to cefotaxime (MIC 8 μg/mL) were observed; the strain remained susceptible to cefepime and imipenem (MIC 0.5 and 0.25 μg/mL, respectively). Clavulanic acid did not restore susceptibility to ceftazidime (MIC 4 μg/mL) but did restore susceptibility to cefotaxime (MIC 0.5 μg/mL). With clavulanic acid, the MIC of cefepime was 0.06 μg/mL.

ESBL in *S. sonnei* is rare worldwide. In Argentina, a CTX-M-2 was found in an isolate of *S. sonnei* resistant to cefotaxime but not to ceftazidime (*4*). In South Korea, TEM-15, TEM-17, TEM-19, TEM-20, TEM-52, and CTX-M-14 were characterized in *S. sonnei* (*5*); TEM-52 and CTX-M-14 were also widely distributed, particularly in *Salmonella* spp. (*6**,**7*)*.* In Turkey, an isolate of *S. sonnei* producing CTX-M-3 was reported (*8*). In Hong Kong, sequencing of 2 *S. sonnei* isolates showed the presence of CTX-M-14 and CTX-M-15 (*9*). Finally, in Bangladesh, 2 isolates of *S. sonnei* with a class A ESBL were reported; they were not characterized at the molecular level, but the resistance phenotypes suggested a CTX-M type (*10*).

In our case, little information on antimicrobial drug resistance could be obtained from Haiti because no systematic investigation on resistance in *Enterobacteriaceae* is performed. Nevertheless, the emergence of TEM-137 (GenBank accession no. AM286274) harbored by this imported *S. sonnei* isolate clearly demonstrates that ESBL-associated shigellosis has emerged in Haiti and that potentially large and severe shigellosis outbreaks could occur, for which the use of azithromycin could be beneficial, as illustrated in our patient. Because treating shigellosis is becoming problematic, it is essential to focus on prevention measures such as simple rules of personal hygiene that might drastically decrease the risk of transmission.
